# Airborne Transmission of a Serotype 4 Fowl Adenovirus in Chickens

**DOI:** 10.3390/v11030262

**Published:** 2019-03-14

**Authors:** Gang Li, Guanliu Yu, Yujuan Niu, Yumei Cai, Sidang Liu

**Affiliations:** 1 College of Animal Science and Technology, Shandong Agricultural University, 61 Daizong Road, Tai’an 271018, Shandong, China; 18854838235@163.com (G.L.); yuguanliu@163.com (G.Y.); yujuanniu@163.com (Y.N.); 2 Shandong Provincial Key Laboratory of Animal Biotechnology and Disease Control and Prevention, Shandong Agricultural University, 61 Daizong Road, Tai’an 271018, Shandong, China; 3 Shandong Provincial Engineering Technology Research Center of Animal Disease Control and Prevention, Shandong Agricultural University, 61 Daizong Road, Tai’an 271018, Shandong, China

**Keywords:** chickens, fowl adenovirus serotype 4, aerosol, hydropericardium syndrome

## Abstract

Serotype 4 fowl adenovirus (FAdV-4) is the main pathogen for hydropericardium syndrome (HPS) in chickens. It has caused major economic losses in the global poultry industry. Currently, FAdV-4′s transmission routes in chickens remain unclear. Here we investigate the airborne transmission routes of FAdV-4 in chickens. A total of 45 ten-day-old chickens were equally divided into three groups (infected group/isolator A, airborne group/isolator B, and control group/isolator C). Of note, isolators A and B were connected by a leak-free pipe. The results showed that the virus could form a viral aerosol, detected in isolators two days post infection (dpi). The viral aerosol reached a peak at 4 dpi in the infected group. Healthy chickens in the airborne group were infected by the virus at 8 dpi. The chickens of the airborne group demonstrated subclinical symptoms capable of shedding the virus for some time. This finding suggests that FAdV-4 can be efficiently transmitted among chickens by aerosol transmission. These findings have significant implications for developing strategies to control this infectious disease epidemic.

## 1. Introduction

Serotype 4 fowl adenovirus (FAdV-4), one of 12 FAdV-4 subtypes (FAdV-1 to 12), is a member of the *Aviadenovirus* genus, *Adenoviridae* family, and contains non-enveloped and double-stranded DNA with a genome of approximately 43–46 kb, the viral genome encodes 10 primary structural proteins (hexon, penton base, fiber, terminal protein, protein µ, protein IIIa, protein V, protein VI, protein VII, and protein VIII) and 11 non-structural proteins [E1A, E1B, E2A (DBP), E3 (ADP), E4, EP, 33 K, 52/55 K, pol, pIVaII, and 100 K] [1,2,3].

Acute avian infectious diseases with hydropericardium syndrome (HPS), inclusion body hepatitis (IBH), and gizzard erosion (GE) are always associated with FAdV infection [4], but most HPS is caused by the FAdV-4 [5]. It has resulted in severe economic losses in the global poultry industry in the last 30 years [6]. In China, there have been a large number of HPS outbreaks caused by the virus in broiler chickens since 2015 [7]. The disease has spread to most of the eastern coastal provinces. It can develop in several breeds of chickens (e.g., broilers, miscellaneous meat-type chickens, Ma chickens, layer chicks and Three-yellow chickens) [8]. Additionally, the virus also infects other birds (e.g., quails, pigeons and wild black kite) [9,10]. Among them, broilers are the main FAdV-4 hosts [11]. HPS can occur in broilers at 3–5 weeks of age with high mortality (up to 80%) [12,13]. Currently, the FAdV-4 epidemic is relatively clear [14,15], but the transmission route varies. Chickens could be infected by poultry vaccines previously contaminated by FAdV-4) [16], by vertical transmission and direct contact [17], or by lateral transmission (the oral–fecal route) [18]. However, to date, the airborne transmission route remains unclear.

According to previous reports, many human viruses that are devastating to animals could form a viral aerosol. These include the Newcastle disease virus (NDV), H9N2 avian influenza virus (H9N2 AIV) [19,20], infectious bronchitis virus (IBV), duck tembusu virus (DTMUV) [21,22], bovine herpes virus 1 (BHV-1) [23], porcine reproductive and respiratory syndrome virus [24,25], and foot-and-mouth disease [26]. Additionally, a subset of HPS viruses could be transmitted by airborne routes such as the quail bronchitis virus [27]. Aerosol plays a great role in spreading these viruses. However, FAdV-4′s potential aerosol transmission in chickens has not been investigated to date. Therefore, to clarify the virus’s route of transmission and effectively control this infectious disease epidemic, in the present study we investigated the airborne transmission of FAdV-4 in chickens.

## 2. Materials and Methods

### 2.1. Ethics Statement

In this study, all the animal experiments with chickens were aperved by the Ethic and Animal Shandong Agricultural University Animal Care and Use Committee (permit number: SDAUA-2015-003)(a). The experiments were performed in accordance with the committee’s established regulations and guidelines.

### 2.2. Experimental Design

**Figure 1:**
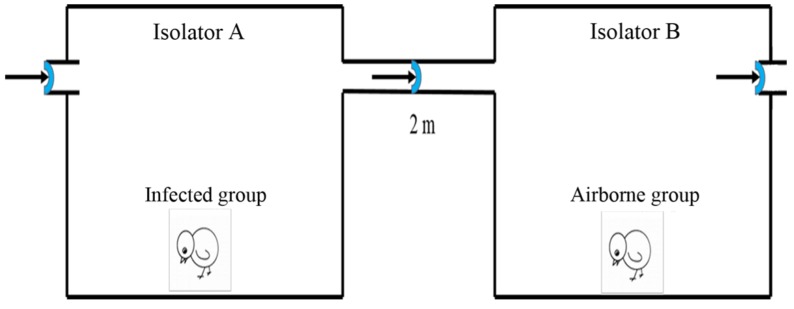
Schematic of FAdV-4 aerosol generation and transmission. Isolator A was a positive pressure isolator, while isolator B was a negative pressure one. The air flowed from isolator A to B.

In the present study, three isolators (isolators A, B, and C) (size: 2.1 m × 0.8 m × 1.5 m; Fengshi Group, Dalian, China) were used as basic equipment. The isolators were cleaned and disinfected thoroughly before beginning the experiments to ensure there were no pathogenic microorganisms. The identical positive–negative pressure isolators A and B were placed in two adjoining rooms. Of note, they were connected by a leak-free pipe (2 m long, 0.08 m diameter) [19]. The air pressure was adjusted to direct airflow from isolator A to B. Incoming air in isolator A (positive pressure isolator) was filtered to avoid contamination by external pathogens. A filter was added between the inoculated and airborne groups. Such a filter could stop the movement of mites or lice between isolators. The air discharged from isolator B (negative pressure isolator) was also effectively filtered to avoid spreading of pathogens and, therefore, guarantee biological safety [28] (Figure 1). Isolator C was placed in an independent house. The isolators’ temperature was suitably adjusted accing to the chickens’ ages. The relative humidity was 30% ± 4% and the airspeed was 0.05–0.20 m/s [29].

A total of 45 ten-day-old specific pathogen-free chickens (Sais Poultry, Jinan, China) were equally divided into three groups (i.e., infected group/isolator A, airborne group/isolator B, and control group/isolator C). The chickens were reared on a net; food and water were autoclaved and refilled automatically. Feces were excreted underneath the net. During the trials’ course, all the chickens’ health status was observed and recorded daily. Dead chickens were necropsied, in a timely manner, in order to perform the pathological examination. All chickens were raised for 16 days and then sacrificed.

### 2.3. FAdV-4 Preparation and Inoculation

The FAdV-4 virus was diluted using Dulbecco’s Modified Eagle Medium (DMEM) (Gibco, Shanghai Ke Biotechnology, Shanghai, China). The infected group was inoculated subcutaneously (s.c.) with 0.18 mL of viral diluent (10^7^ TCID50/chicken) of FAdV-4 strain SDDM-4/15. In a previous study from our group, we analyzed this virus in detail [8]. The chickens in the airborne and control groups were inoculated with 0.18 mL DMEM/chicken.

### 2.4. Clinical Observation and Post-Mortem Examination

In the course of the study, we observed the chickens’ health status. Dead chickens were necropsied to examine pathological changes during the trial. Furthermore, all chickens were euthanized after the study to detect the virus in the livers by polymerase chain reaction (PCR). FAdV-4 positive liver tissues were used to extract viral DNA. Two fragments of Hexon gene were amplified by PCR with specific primers (Table 1). The PCR was conducted in a reaction volume of 50 μL containing 3 μL viral genomic DNA, MgCl_2_ (8 mmol/L), 2 μL of dNTP (0.2 mmol/L), and each primer (100 mmol/L) contained 5 μL 10 × PCR buffer and 0.25 μL Ex Taq DNA polymerase (Takara, Dalian, China). The amplification programs were performed according to the following protocol: 94 °C for 5 min, followed by 32 cycles of 94 °C for 50 s, 56 °C for 60 s, 72 °C for 100 s, and a final elongation step of 8 min at 72 °C. PCR products were analyzed by a 0.9% (*w*/*v*) agarose gel electrophoresis. Positive PCR products were subsequently purified and cloned into pEASY-T1 vector (TaKaRa, Dalian, China) according to the manufacturer’s instructions. Positive clones were then sequenced using the Sanger dideoxy sequencing method (Sangon Biotech, Shanghai, China).

### 2.5. Collection of Air Sample

Before the beginning of the trial, the air samples in isolators A, B, and C were collected and evaluated to guarantee the isolators were free of pathogenic contamination. Subsequently, air samples in all isolators were collected by AGI-30 (All Glass Impinger, Liaoyang, China). Collection was performed at a flow rate of 12.5 L/mL for 40 min at 2, 4, 8, 12, and 16 dpi [28]. We used 50 mL sterilized phosphate buffer solution (PBS. pH 7.2, containing 1000 U/mL penicillin, 1 mg/mL streptomycin) as a sampling medium. All samples were stored at 4 °C and used within 24 h. Air collection fluid was first spun at 8000× *g* for 30 min to remove the pelleted bacteria and dust. It was then centrifuged at 100,000× *g* for 2 h to collect the viral pellet. The viral pellet was diluted with 0.6 mL PBS. Viral DNA was extracted by a DNeasy Tissue kit (Qiagen, Hilden, Germany) to detect the concentration of FAdV-4 aerosol in the isolators. Quantification was performed by qualitative real-time PCR (qPCR) with specific primers (Table 1). qPCR was performed using the UltraSYBR Mixture (High ROX) (CWBIO, Beijing, China) and the ABI PRISM 7500 Real-time PCR System (Applied Biosystems, Foster City, CA, USA). The qPCR was conducted in a total volume of 20 μL, following the manufacturer’s instructions. The amplification steps were set as follows: stage 1, 95 °C for 10 min, stage 2, 40 cycles of denaturation at 95 °C for 15 s and at 60 °C for 60 s, followed by a dissociation curve analysis. Each sample was analyzed in triplicate.

### 2.6. Collection of Serum, Liver, Oral, and Cloaca Samples

In this study, in order to confirm the presence of infectious FAdV-4, we detected the viremia and the FAdV-4 positive rate of the liver. Five blood samples were obtained randomly from the jugular vein of the chickens in each group at 0, 2, 4, 8, 12, and 16 dpi. The serums were separated from the blood samples by centrifugation. A portion of the product was used to detect FAdV-4 load by qPCR. The remainder was used to examine antibody responses against FAdV-4 by enzyme-linked immunosorbent assay kit (Biochek USA Corporation, Scarborough, ME, USA).

Ten oral and cloacal swabs were randomly collected from each group at 0, 2, 4, 8, 12, and 16 dpi. The swabs were used to identify the virus’ shedding by PCR.

### 2.7. Hexon Gene Sequence Alignment

In the present study, the Clustal W method in the MegAlign program of DNAStar was used to further confirm airborne transmission of the virus. This method allowed the alignment of amplified fragments (2524 bp, linked by the two fragments) of FAdV-4′s *Hexon* gene in the virus-positive liver of chickens between the infected group and the airborne group.

## 3. Results

### 3.1. Clinical Observations and Necropsy/Post-Mortem Findings

**Figure 2:**
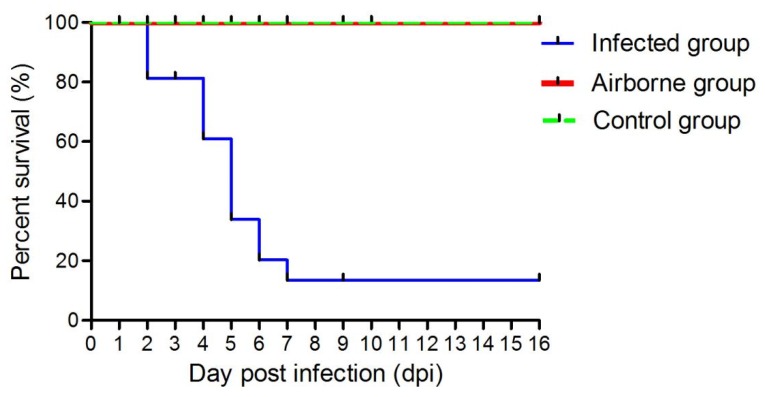
Survival curve analysis of experimental chickens at different time points post infection. Note: the green line (airborne group) is not obvious because it overlaps with the black line (control group).

In the infected group, the chickens showed signs of depression after 1 dpi. Additionally, three died at 2 dpi, three at 4 dpi, four at 5 dpi, two at 6 dpi, and one at 7 dpi. The infected group’s overall mortality was 86.7%. Necropsies of the dead chickens revealed visible pericardial effusion and liver necrosis (figure not shown). No obvious pathological changes were observed in the chickens of the airborne and control groups (figure not shown). We did not record any death in the airborne and control groups until the experiment ended (Figure 2).

### 3.2. Detection of FAdV-4 Aerosol

**Figure 3:**
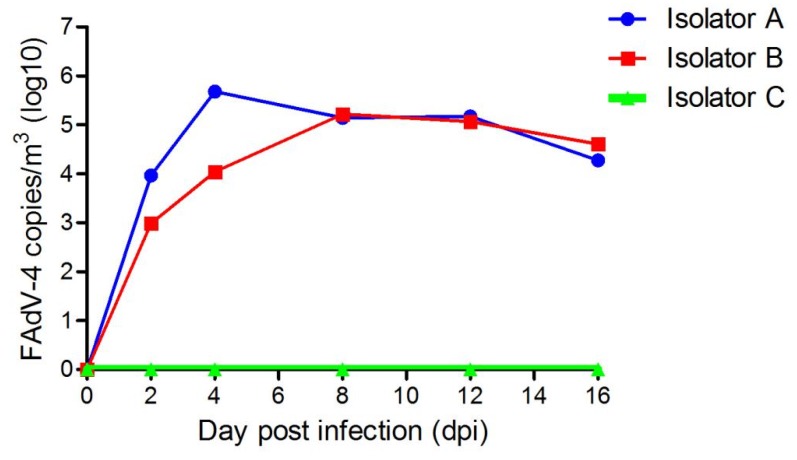
The concentration of the FAdV-4 aerosols in isolator A and B at different time points post infection.

In this study, the FAdV-4′s aerosol was detected in isolator A at 2 dpi, at a concentration of 9.16 × 10^3^ copies/m^3^. The maximum concentration of the viral aerosol recorded was 4.75 × 10^5^ copies/m^3^ at 4 dpi. It remained there until the end of the trial. In isolator B, the concentration of viral aerosol was 9.68 × 10^2^ copies/m^3^ at 2 dpi. Its maximum concentration was 1.64 × 10^5^ copies/m^3^ at 8 dpi. Moreover, the virus aerosol concentrations kept increasing, reaching the same level as isolator A at 16 dpi (Figure 3). No viral aerosol was detected in control group.

### 3.3. Viremia and FAdV-4 Antibody

**Figure 4:**
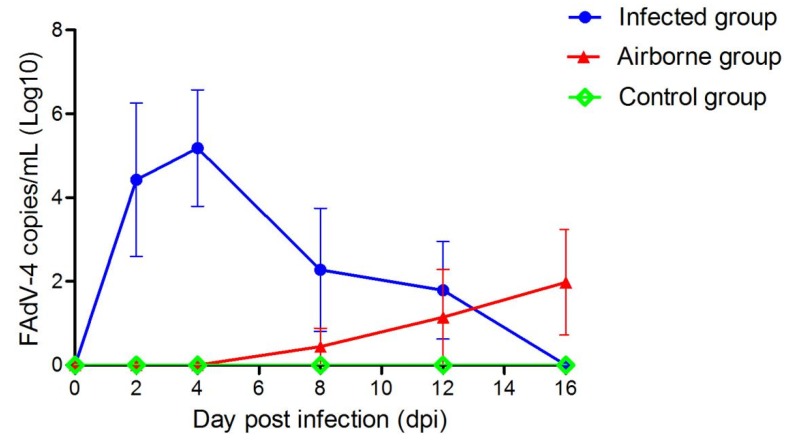
Viral genome in the serum of chickens at different time points. The data were presented as the mean ± standard deviation.

**Figure 5:**
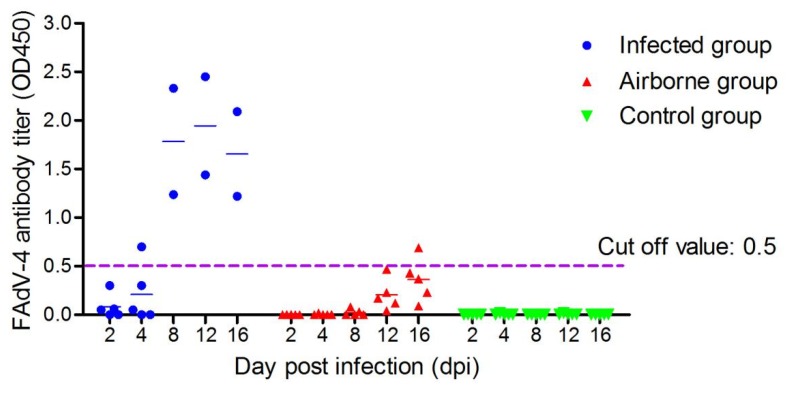
Antibody titers. Values were higher than 0.5, indicating that the serum antibody was positive.

Five chickens per group were tested for viremia and antibody at each time point. In the infected group, viremia occurred at 2 dpi and lasted until 16 dpi. In the airborne group, viremia was detected in one chicken at 8 dpi and another one at 12 dpi. Additionally, two chickens showed viremia at 16 dpi (Figure 4). Seroconversion was detected in the chickens at 4 dpi (Figure 5). Chickens in the infected group showed the highest antibody level at 12 dpi. In the airborne group, only one chicken showed seroconversion at 16 dpi. Neither viremia nor antibody titers were observed in the control group.

### 3.4. Virus Shedding

**Figure 6:**
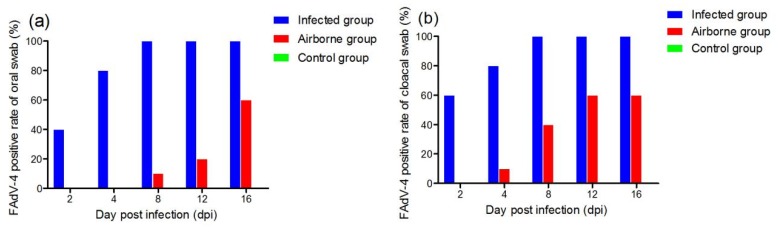
FAdV-4 positive rates of oral swab (**a**) and cloacal swab (**b**) at different days post infection.

**Figure 7:**
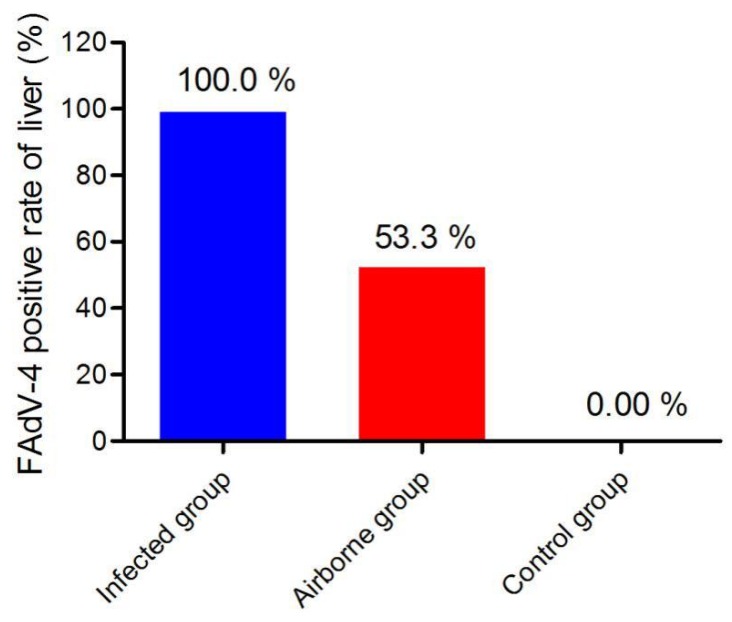
FAdV-4 positive rates of liver samples in different groups. Notes: liver samples of each group were collected from dead or healthy chickens from 2 dpi to 16 dpi.

To detect virus shedding in the surviving chickens in all groups, oral and cloacal swabs were collected from 2 to 16 dpi and examined by PCR. In the infected group, shedding of viruses appeared in 40% (4/10) and 60% (6/10) of oral and cloacal swabs at 2 dpi. Additionally, all chickens (2/2) shed viruses from 8 to 16 dpi. However, in the airborne group, 10% (1/10) of the cloacae swabs was viral DNA-positive at 4 dpi, and at 16 dpi, 60% (6/10) chickens were positive. No viral DNA-positive was detected in the control group (Figure 6 and Figure 7).

### 3.5. Viral DNA Detection in the Liver

As shown in Figure 7, FAdV-4 positive rate in the liver reached up to 100% (15/15) in the infected group. In the airborne group, 53.3% (8/15) of the liver samples were infected by FAdV-4. No viral DNA was detected in the control group.

In the infected group, viral DNA was detected in all (15/15, 100%) tissue samples collected from dead or live chickens. In the airborne group, 53.3% (8/15) of the liver samples collected from the sacrificed chickens were positive for viral DNA. No viral DNA-positive was detected in the control group.

### 3.6. Sequence Alignment of Hexon Gene

In this study, the *Hexon* gene sequence analysis revealed 99.9% sequence identity (at the nucleotide level) between the infected group isolate and the airborne group strain. This result provided sufficient evidence to further confirm that the virus in the airborne group originated from the infected group.

## 4. Discussion

FAdV-4, a non-enveloped, double-stranded DNA virus, plays a major role in the pathogenesis of HPS in broilers aged 3–5 weeks [30]. The disease was first reported in Angara Goth (also named Angara disease), Pakistan, in 1987. There were subsequent outbreaks in India, South Asia, China, Japan, South Korea, Eastern Europe, Mexico, and North America. It has caused huge economic losses to the poultry industry worldwide [31]. To effectively control the spread and epidemic of this infectious disease, an exhaustive study of FAdV-4′s transmission route is required.

To the best of our knowledge, this is the first study to evaluate airborne transmission of FAdV-4. During the trials, dynamic monitoring of FAdV-4 aerosol generation and transmission revealed FAdV-4 in air samples from isolators A, B, and C. FAdV-4 aerosol concentration of isolators A and B peaked at 4.75 × 10^5^ copies/m^3^ air at 4 dpi, and 1.64 × 10^5^ copies/m^3^ air at 8 dpi, respectively. Both remained at higher concentrations for a long time. Such findings indicate that FAdV-4′s aerosol model generation and transmission was successfully established.

We observed a positive correlation between the concentrations of virus aerosols and the number of infected chickens [32]. However, in the present study, as the number of infected live chickens decreased (Figure 1), the concentration of the viral aerosol of the infected group did not reduce (Figure 2). This could be caused by the virus released from feces into the air. Furthermore, the aerosol concentration was also linked to the body weight of the chickens and the accumulation of aerosol. Additional influencing factors were temperature [33], speed of air current [34], the chickens’ activity, and herd density [35].

In the present study, the viral aerosol in the A and B isolators could be detected at 2 dpi, which indicated that the virus could proliferate rapidly in the air of isolators. The virus excreted by oral and cloaca of infected chickens, other transmission routes (e.g., contact transmission) also accelerate the spread of FAdV-4 in chickens. However, detection of the viraemia and FAdV-4 positive antibody of chickens in the airborne group occurred at 8 and 16 dpi respectively. Of note, these time points were significantly later than those in the infected group (i.e., 2 and 8 dpi respectively) (Figure 3 and Figure 4). These different time points may be due to the lower concentration of FAdV-4 aerosol in the airborne group of 8 days ago (Figure 2). The lower the viral aerosols’ concentration, the less likely the chickens were to be infected, consistent with the above phenomenon.

Here, based on FAdV-4′s aerosol concentration indices, FAdV-4 antibody titer, viremia, sequence alignment, FAdV-4 positive rates of oral swab, cloacal swab, and liver in the airborne group, we can conclude that the chickens in the airborne group have been infected by FAdV-4 aerosol originating from the infected group. However, the chickens didn’t show significant pathological lesions (e.g., higher antibody titer or mortality rate). This may be due to the development of a subclinical infection, which usually does not show any clinical feature, and can only be found by immunological detection [36]. Of note, infections pose greater potential risks for the poultry industry because virus infected fowls are still alive and could continue to excrete the virus into the surroundings through the feces or oral cavity.

## 5. Conclusions

In conclusion, our findings provide adequate evidence that FAdV-4 could spread by aerosols in chickens. Thus, regular ventilation and disinfection in the breeding environment are critical measures to block FAdV-4′s spread.

## Figures and Tables

**Table 1:** Primers used in the study.

Primer Name	Sequence(5′-3′)	Product Size (bp)	GenBank No.
FAdV (qPCR)	F: 5′-GACGGCGGCGCAGGTGACGAAGATT-3′	126	U46933
	R: 5′-TGAGACTTGGCGAAGCGACCGAGCA-3′		
FAdV (PCR)	F: 5′-AATTTCGACCCCATGACGCGCCAGG-3′	508	
	R: 5′-TGGCGA AAGGCGTACGGAAGTAAGC-3′		
FAdV (Hexon)	F1: 5′-TGGACATGGGGGCGACCTA-3′	1219	
	R1: 5′-AAGGGATTGACGTTGTCCA-3′		
	F1: 5′-AACGTCAATCCCTTCAACCACC-3′	1350	
	R1: 5′-TTGCCTGTGGCGAAAGGCG-3′		
